# Systematic review and meta-analysis of the additional benefit of local prophylactic antibiotic therapy for infection rates in open tibia fractures treated with intramedullary nailing

**DOI:** 10.1007/s00264-014-2293-2

**Published:** 2014-02-15

**Authors:** Joyce Craig, Thomas Fuchs, Michelle Jenks, Kelly Fleetwood, Dominik Franz, Joel Iff, Michael Raschke

**Affiliations:** 1 York Health Economics Consortium, University of York, York, YO10 5NH UK; 2 Department of Trauma, Hand and Reconstructive Surgery, University Hospital Muenster, Waldeyerstrasse 1, 48149 Muenster, Germany; 3 Quantics Consulting Ltd, Hudson House, 8 Albany Street, Edinburgh, EH1 3QB UK; 4 Department of Medical Management/Medical Controlling, DRG-Research-Group, University Hospital Muenster, Domagkstrasse 20, 48149 Muenster, Germany; 5 Synthes GmbH, Luzernstrasse 21, 4528 Zuchwil, Switzerland

**Keywords:** Tibia fracture, Local antibiotics, Infection

## Abstract

**Purpose:**

This analysis compared the rate of deep wound infections in patients with open tibia fractures, treated with intramedullary nails, receiving additional locally-delivered antibiotics to those receiving standard care.

**Methods:**

Two systematic literature searches identified studies reporting infection rates in patients treated with intramedullary nails for tibia fractures receiving systemic antibiotics only (search one) and in patients receiving adjunctive locally-administered antibiotics peri-operatively at the tissue-implant interface (search two). After applying inclusion and exclusion criteria, 14 and seven papers from searches one and two, respectively, were included in meta-analyses.

**Results:**

The absolute rate of infection was lower for all Gustilo-Anderson grades of tibia fractures when local antibiotics were administered as adjunctive prophylactic therapy. For severe fractures, classified as GAIII fractures, patients receiving systemic antibiotics only had an infection rate of 14.4 % [95 % CI: 10.5 %, 18.5 %]; adding local antibiotics reduced the rate to 2.4 % [0.0 %, 9.4 %], with an odds ratio of 0.17. Risk of deep wound infections increased with severity of fracture, rising to over 31 % in GIIIB&C fractures for patients receiving systematic antibiotics only, but to below 9 % with additional local antibiotics.

**Conclusion:**

The findings support consideration of augmenting the antibiotic prophylaxis regimen to include locally-delivered antibiotics. Patients with severe fractures will obtain greatest benefit from infections avoided. No trial directly compared the two treatments for open tibia fractures, limiting the ability to attribute the differences in observed infection rates directly to the treatments themselves. A large comparative study to improve the evidence on relative effect size is merited.

Level of evidence: Level III.

## Introduction

Open tibia fractures with severe soft-tissue damage and disrupted vascularity are especially prone to infection. Despite improved treatments, better surgical techniques and prophylactic treatment with systemic antibiotics, deep wound infections still occur and can lead to osteomyelitis, reduced limb function, increased disability and life threatening septic conditions. The risk of infection is related to severity of trauma, condition of the local environment including skin loss, and immunocompromised patients, for example, those with chronic disease, obese or smokers [1]. From the hospital’s perspective affected patients have additional surgery and medication, as well as a prolonged length of stay, with associated higher costs, e.g. the patient has chronic pain, a higher risk of disability, including amputation, failure to achieve fracture healing and reduced quality of life. A recent systematic review by Papakostidis et al. [2] reported deep infection rate by treatment type and grade of fracture. The grading system adopted was the Gustilo-Anderson (GA) classification which describes soft tissue injury [1]. Patients treated with intramedullary nails had lower deep wound infection rates reported compared to those treated by external fixation or plating [2]. Materially different wound grade infection rates were also reported, rising from 1.7 % for GA grade 1 fractures to 9.2 % for those with GA grade IIIB treated with intramedullary nails and from 1.8 % to 12.3 % for all treatments [2].

Systematic review evidence reported that prophylactic administration of systemic antibiotics was associated with a 60 % reduction in absolute risk of early wound infections compared with no administration or placebo [3]. This is now accepted as standard practice to control bacterial contamination and reduce infection after surgery.

Clinicians continue to innovate to find ways to prevent and treat infections, thereby improving standard care. One approach is to increase the effectiveness of antibiotics particularly by trialling systems to deliver antibiotics at the tissue–implant interface [4–9]. Placing antibiotics at the implant site may prevent bacteria from colonising the implant surface and then forming a biofilm shield, limiting the action of systemic antibiotics.

The objective of this review and meta-analysis [10] was measuring the additional benefit to patients with open tibia fractures treated with intramedullary nails through adding locally-delivered, as adjunct to, systemic antibiotics. The outcome measure adopted was deep wound infections avoided. The study group was restricted to patients treated with intramedullary nails because they already have the lowest existing infections rates [2], are preferred use in standard care [1] and provides the largest evidence base. Analysis was by fracture grade, capturing the effect of soft tissue damage severity.

## Methods

### Literature search

Two literature searches were conducted:

- Search one updated the earlier systematic review [2] with studies reporting on infection rates in patients treated with intramedullary nails for tibia fractures, receiving systemic antibiotics only, and limited to studies published from 2009 to 22nd November 2012.
- Search two identified studies reporting infection rates for patients receiving adjunctive locally-administered antibiotics peri-operatively at the tissue–implant interface, and was limited to studies published from 1980 to 22nd November 2012.

Search strategies were devised using a combination of subject indexing terms such as Medical Subject Headings (MeSH) in MEDLINE, and free text search terms in the title and abstract. Strategies adopted for Ovid MEDLINE(R) are available as additional material from the corresponding author, together with associated protocol, statistical plans and evidence tables. The databases and information sources searched were: MEDLINE and MEDLINE In-Process; EMBASE; Science Citation Index (SCI); Cochrane Database of Systematic Reviews (CDSR); Cochrane Central Register of Controlled Trials (CENTRAL); DARE Database of Abstracts of Reviews of Effects (DARE); Health Technology Assessment Database (HTA); ClinicalTrials.gov; International Clinical Trials Registry Platform (ICTRP); and MetaRegister of Controlled Trials (mRCT). The searches were supplemented by hand searching.

### Inclusion criteria, selection, data extraction and grading

Search one’s main inclusion criteria concerned at least 50 patients with open tibial fractures treated with intramedullary nails, reporting deep wound infection rates. For search two, criteria were extended to include patients receiving prophylactic antibiotics at the tissue–implant interface. No size limit was applied but a language restriction of English and German was adopted. Deep wound infections included in this study are defined using the criteria set out in the Centers for Disease Control and Prevention document 'Definition of Healthcare-Associated Infection and Criteria for Specific Types of Infections in the Acute Care Setting' [11]. Where studies did not describe infection according to these criteria, judgements on inclusion were taken. Titles and abstracts of all papers found were assessed. Full papers were retrieved if they appeared to meet the inclusion criteria; they were then read and included if they did.

**Fig. 1:**
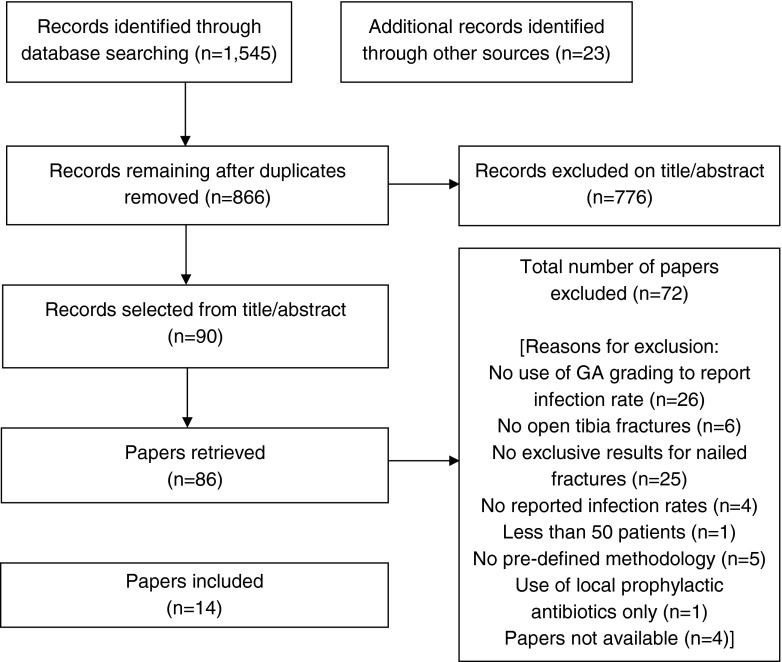
PRISMA flow diagram search one. (Search one updated the earlier systematic review [2] with studies reporting on infection rates in patients treated with intramedullary nails for tibia fractures and receiving systemic antibiotics only)

**Fig. 2:**
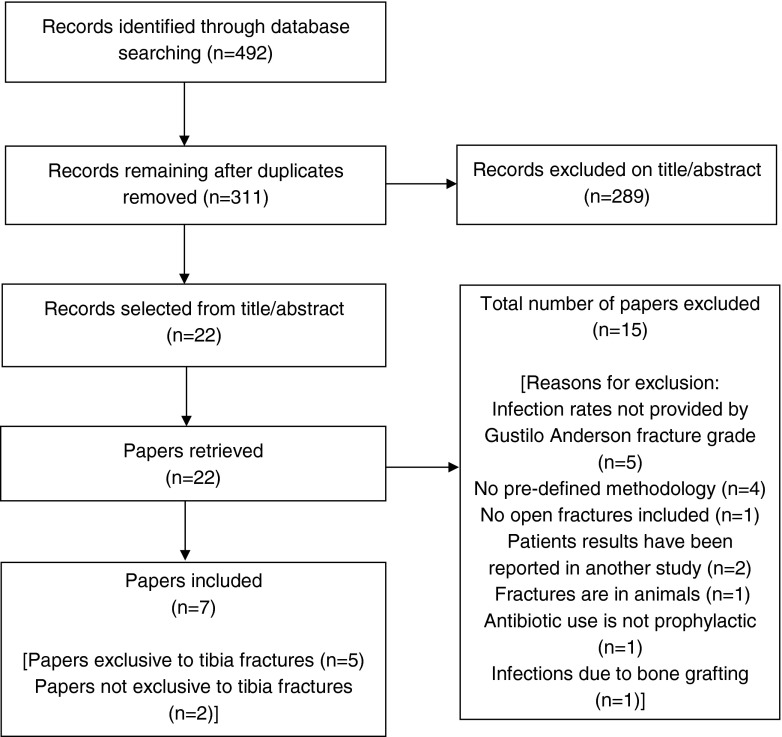
PRISMA flow diagram search two. (Search two identified studies reporting infection rates for patients receiving adjunctive locally administered antibiotics perioperatively at the tissue–implant interface)

PRISMA diagrams are provided in Figs. 1 and 2 to show number of papers retrieved and excluded, together with reasons, at various stages (Figs. 1 and 2). For search one, 14 papers were included which reported infection rates by GA grade when all antibiotics were systemic. Of these, eight were retrospective reviews [8, 12–18], five were prospective randomised studies [19–23] and one was a review of studies [24]. Of the seven papers identified in search two reporting infection rates with locally-administered antibiotics, six were retrospective reviews or case studies [4, 5, 7–9, 25] and one was a randomised study [6]. One paper [8] was included in both groups. Authors of the largest study [23] of 1,226 patients were contacted and provided additional data beyond that published.

Detailed evidence tables, prepared by one person and checked by a second, described the study aims, patients, methods and results for each included study. Each study’s quality of evidence was graded using the GRADE process [26]. This has four categories of evidence—high, moderate, low and very low—and provides a measure of the risk of bias. Thirteen studies were observational and graded low [4, 5, 7–9, 12–18, 25], five were moderate [6, 19–22, 24] and one high [23].

No studies directly compared patients with tibia fractures plus locally-delivered antibiotics to patients with systemic antibiotics only. Most studies of locally-delivered antibiotics [4–8] used polymethylmethacrylate bead chains impregnated with vancomycin or tobramycin. These were placed directly on the fracture site during the peri-operative period and removed as healing progressed. Two studies [9, 25] used a gentamicin-loaded coating on an intramedullary nail. Systemically administered antibiotics included cefazolin, cefuroxime, meronem, tobramycin, gentacimin and penicillin. Where reported, all patients receiving both local and systemic antibiotics were given at least two different antibiotics.

### Meta-analyses

None of the standard methods for meta-analysis of proportions easily handle studies with rates close to zero; hence, the arcsine transformation was adopted. Fixed and random effects models were used. For the random effects model the between study variance (τ^2^) was estimated using the restricted maximum likelihood method. All studies, although of poor quality, were judged sufficiently homogeneous in terms of patients, interventions, outcomes, settings and study design to merit attempting meta-analysis to provide greater statistical power to inform treatment effects. However, no randomised control trials, the highest level of evidence, were included within the meta-analysis which limits the interpretation of the results. Only random effects models are presented, being more appropriate if the studies are clinically and methodologically heterogeneous. The I^2^ statistic which measures the percentage of variation across studies that is due to heterogeneity is reported. Confidence intervals were calculated according to the Clopper and Pearson method [27]. In analyses with very low infection rates the lower confidence limit was set to zero and should be considered approximate only. All analyses were conducted using R statistical software. Infection rates for five groups were calculated: GAI, GAII, GAIIIA, GAIIIB/C and all GAIII fractures combined. Grades B and C were combined because there were few cases of GAIIIC fractures reported.

## Results

Results presented in Tables 1 and 2 show the absolute rate of infection is lower for all GA grades when local antibiotics are administered as adjunctive prophylactic therapy. For example, for all GAIII fractures, those with systemic antibiotics only had an infection rate of 14.4 % [10.5 %, 18.5 %]; with addition of local antibiotics the rate was 2.4 % [0.0 %, 9.4 %], an odds ratio of 0.17.

**Table 1:** Deep wound infections in open tibia fractures treated with intramedullary nails; systemic antibiotics only

Fracture grade	Number of studies [References]	Number of infections	Number of fractures	Random effects result (estimate [95 % CI]) %	I2
GAI	9 [8, 12–16, 21–23]	18	469	1.59 [0.18, 4.36]	60 %
GAII	10 [8, 12–17, 21–23]	33	510	2.99 [0.66, 6.92]	70 %
GAIII AB&C	12 [8, 12, 14, 16–24]	101	702	14.36 [10.47, 18.75]	62 %
GAIII A	7 [8, 12, 14, 16, 17, 19, 23]	31	186	11.24 [3.82, 21.91]	64 %
GAIII B&C	6 [8, 12, 14, 15, 17, 23]	25	109	31.18 [7.82, 61.51]	78 %

**Table 2:** Deep wound infections in open tibia fractures treated with intramedullary nails; with adjunctive locally-delivered antibiotics

Fracture grade	Number of studies [References]	Number of infections	Number of fractures	Random effects result (estimate [95 % CI]) %	I2
GAI	2 [6, 9]	0	17	0.00 [0.00, 5.54]^a^	0 %
GAII	3 [4, 6, 9]	2	46	2.25 [0.00, 12.01]^a^	6 %
GAIII AB&C	5 [4, 6, 8, 9, 25]	3	75	2.41 [0.00, 9.41]	20 %
GAIII A	2 [4, 6]	0	43	0.00 [0.00, 2.22]^a^	0 %
GAIII B&C	5 [4, 6, 8, 9, 25]	3	28	8.76 [1.32, 21.80]	0 %

^a^Low infection rate. Confidence interval is approximate only

In both groups, risk of deep wound infections increased with the severity of soft tissue injury, rising to over 31 % in GIIIB&C with those receiving systematic antibiotics only but to under 9 % when antibiotics were delivered directly at the implant site. The weighted mean follow-up time post treatment, where reported, for patients receiving systemic antibiotics only was 15.1 months and for those receiving additional local antibiotics was 19.2 months. The duration of systemic antibiotic treatment upon hospital admission was inconsistently reported.

Recognising that sample size for the number of fractures benefiting from locally-delivered antibiotics is small, i.e. only 75 GAIII grade fractures, inclusion criteria were relaxed to include patients with any long bone open fracture treated by any method. Two large studies were now included, with Ostermann et al. [28] providing 139 upper limb fractures and 706 in the lower limbs and Henry et al. [5] having 23 upper limb fractures and 204 lower limb fractures. Both were retrospective reviews and graded ‘low’. The weighted mean follow-up time with additional long bone fracture studies included was 19.1 months. Infection rates of patients treated with local antibiotics were similar for all fractures/all treatments (Table 3) and with tibia fractures except those with most severe injuries. Infection rate for all fractures/treatments was 5.9 %, 33 % lower than patients with tibia fractures (8.8 %). This is consistent with open tibia fractures having more extensive comminution, segmental bone loss and poorer vascularisation than other long bone fractures, thereby increasing the risk of infection [29, 30].

**Table 3:** Deep wound infections in long-bone fractures treated with antibiotics delivered at the tissue-implant interface

Grade	Number of studies [References]	Number of infections	Number of fractures	Random effects result (estimate [95 % CI]) %
GAI	4 [5, 6, 9, 28]	0	245	0.35 [0.00, 1.48]^a^
GAII	5 [4, 5, 6, 9, 28]	2	425	2.45 [1.19, 4.13]
GAIII AB&C	7 [4, 5, 6, 8, 9, 25, 28]	3	547	3.92 [2.45, 5.70]
GAIII A	4 [4, 5, 6, 28]	0	250	1.42 [0.17, 3.89]
GAIII B&C	7 [4, 5, 6, 8, 9, 25, 28]	3	293	5.91 [3.50, 8.89]

^a^Low infection rate. Confidence interval is approximate only

No adverse events were reported from use of locally-delivered antibiotics.

## Discussion

A comparison of the results from the recent systematic review [2] shows consistency of effect size for low grade fractures where rates of 1.7 % for GA1, 3.1 % for GAII and 2.4 % for GAIIA were identified. Conversely, for more severe fractures the present review identified an infection rate of over 31 % in grade B&C fractures compared to the published rate of 9.2 % for all grade B fractures [2]. Part of the difference can be explained because Papakostidis [2] included studies using locally administered antibiotics and these studies reduced the mean infection rate.

Meta-analyses identified that patients who received locally-delivered antibiotics as prophylaxis, in addition to systemic antibiotics, had materially lower infection rates that those receiving standard systemic antibiotics. For the most severe case (GAIII B&C) the incidence of infections fell from over 31 % with systemic antibiotics only to under 9 % with the addition of local antibiotics. Given the severe consequences for patients and healthcare systems of such infections, the findings support consideration of augmenting the antibiotic prophylaxis regimen to include locally-delivered antibiotics. This is not likely to influence the rate of resistance because of the locally very high concentration level and high release rate when locally delivering antibiotics, thereby reducing the exposure to sub-inhibitory concentrations. An increased risk may be considered when antibiotic-loaded PMMA beads are implanted and not removed at the appropriate time point.

Fifteen of the 21 papers forming the evidence base to support this conclusion were graded low, with a risk of bias in the results’ precision; five were graded of moderate quality, consistent with moderate confidence in the effect size, and one, a large multi-centred randomised controlled trial was graded high.

No size limit was applied to studies of the locally-delivered antibiotics. Three of the seven papers included had less than 30 patients and hence had a relative lack of power to detect events, particular adverse events. As a counterbalance one study included over 900 fractures [28] and a second 227 fractures [5].

Absence of a directly comparable group of matched patients limits the ability to attribute the differences in observed infection rates directly to the treatments themselves. Different infection rates could arise from differences in patient or study treatments that may influence infection rate. No adjustment could be made for case-mix due to insufficient information reported in included studies. Notably, among the papers included in our analysis, we were unable to determine retrospectively the duration of prophylactic antibiotic administration, the choice of prophylactic antibiotic agents, or the pathogens of further infections. This is the main limitation of the literature review. However, it is reassuring that all studies from a range of geographical settings provide consistent infection rates. The poor quality of the papers further limits the reliability of the results of the meta-analysis. Uncertainty exists in certain papers around the grade of infections because not all papers reported the classification adopted. The reported infection grades were used and a check was undertaken to identify consistency with other studies. However there is always a risk of inconsistent definitions.

The observed benefit of locally-delivered antibiotics in preventing deep wound infections is judged sufficiently large, particularly for patients with more severe fractures, that the conclusion is judged robust despite these weaknesses. A large comparative study would be merited to confirm findings and provide certainty on effect size.
